# Common pathways to Dean of Medicine at U.S. medical schools

**DOI:** 10.1371/journal.pone.0249078

**Published:** 2021-03-25

**Authors:** Clare E. Jacobson, Whitney H. Beeler, Kent A. Griffith, Terence R. Flotte, Carrie L. Byington, Reshma Jagsi

**Affiliations:** 1 University of Michigan Medical School, Ann Arbor, MI, United States of America; 2 Department of Radiation Oncology, University of Michigan, Ann Arbor, MI, United States of America; 3 The Center for Cancer Biostatistics, University of Michigan, Ann Arbor, MI, United States of America; 4 School of Medicine, University of Massachusetts Medical School, Massachusetts, United States of America; 5 University of California Health System, University of California, Oakland, CA, United States of America; 6 Department of Pediatrics, UCSF, San Francisco, CA, United States of America; 7 Department of Radiation Oncology, University of Michigan, Ann Arbor, MI, United States of America; 8 Center for Bioethics and Social Sciences in Medicine, University of Michigan, Ann Arbor, MI, United States of America; SUNY Downstate: SUNY Downstate Health Sciences University, UNITED STATES

## Abstract

**Purpose:**

We sought to evaluate common leadership experiences and academic achievements obtained by current U.S. Medical School Deans of Medicine (DOMs) prior to their first appointment as Dean in order to elucidate a common pathway for promotion.

**Methods:**

In April-June 2019 the authors requested a curriculum vitae from each of the 153 LCME-accredited U.S. Medical School DOMs. The authors abstracted data on prior appointments, demographics, and achievements from CVs and online databases. Differences by gender and institutional rank were then evaluated by the Fisher’s exact and Wilcoxon rank sum tests.

**Results:**

CVs were obtained for 62% of DOMs (95 of 153), with women comprising 16% of the responding cohort (15/95). Prior to appointment as DOM, 34% of respondents had served as both permanent Department Chair and Associate Dean, 39% as permanent Department Chair but not Associate Dean, and 17% as Associate Deans but not permanent Department Chair. There was a non-significant trend for men to have been more likely to have been a permanent Department Chair (76% vs 53%, p = 0.11) and less likely to have been an Associate Dean (48% vs 67%, p = 0.26) compared to women. Responding DOMs at Top-25 research institutions were mostly male (15/16), more likely to have been appointed before 2010 (38% vs 14%, p = 0.025), and had higher H-indices (mean (SD): 73.1 (32.3) vs 33.5 (22.5), p<0.01) than non-Top-25 Deans.

**Conclusions:**

The most common pathway to DOM in this study cohort was prior service as Department Chair. This suggests that diversification among Department Chair positions or expansion of search criteria to seek leaders from pools other than Department Chairs may facilitate increased diversity, equity, and inclusion among DOM overall.

## Introduction

Little research has systematically described the prerequisite achievements and experiences expected of the Deans of Medicine (DOMs) who constitute the very top echelon of academic medicine’s hierarchy and have unique influence over the direction of their institutions and the profession more generally. Similarly, little is known about whether these academic stepping stones might differ according to gender or institutional rank. Previous work has drawn attention to the delays women face in promotion to DOM and called for further elucidation of a “common pathway” in order to expedite their advancement (and presumably that of others with minority identities) into these influential positions [1]. At present, however, there are limited and conflicting data regarding which academic leadership positions most commonly lead to Dean, or whether these differ by gender or the Medical School’s national rank [2, 3].

The proportion of women decreases as one ascends the ranks of academic medicine [2, 4–7]. In 2019, for example, women represented 52% of matriculating U.S. medical students, yet only 26% of full professors, 19% of department chairs, and 18% of Medical School Deans [8–10]. Within decanal offices, women are more prevalent in lower tiered leadership positions, with more than threefold higher representation among Assistant Dean compared to DOM positions [2].

Given increased interest and attention on diversifying academic medicine, it seems imperative to understand who is at the top of these institutional hierarchies and how they got there [11]. Diversity in leadership may yield important consequential benefits. Indeed, the 2018 NASEM report on sexual harassment suggests that “given the critical role that leaders play in setting the tone of organizational culture and the significance of their identity, it is plausible to suggest that more women of color and persons with minority ethnic, gender, and sexual identities in leadership positions will reduce the likelihood of sexual harassment in academic institutions [12].” Diversity in leadership may also improve an institution’s financial performance, as well as the quality of research and patient care [13].

In this study, we sought to evaluate common leadership experiences and other metrics of academic achievement held by current U.S. Medical School DOMs prior to their first appointment as Dean. We then sought to compare these qualifications according to DOM gender and medical school ranking, hypothesizing that women may be disproportionately likely to take routes to deanship other than by being Department Chair. By identifying, illuminating, and disseminating this information, we aim to inform efforts to promote equal opportunity and access to the full breadth of candidates qualified to assume these pivotal leadership positions.

## Methods

This was a prospective, cross-sectional study performed in April—June 2019. The study was deemed exempt from review by the University of Michigan’s IRB committee. We requested a current curriculum vitae (CV), resume, or biosketch from each of the 153 LCME-accredited U.S. Medical School DOMs as of February 2019 (all schools with full, preliminary, and provisional accreditation statuses were included, and all DOMs—whether permanent, acting, or interim, were included). CVs were directly uploaded and stored within a secure research database accessed only by the study team. Deans were invited to participate in person at the March 2019 Council of Deans meeting in New Orleans, LA, as well as over the phone and by email communication with each school’s official Dean’s Office. Executive and Administrative Assistants were engaged in this process in order to increase response rates and lessen the burden on participating Deans. CVs that were publicly available online were also included in the cohort; we used submitted CVs over public CVs when both were available.

From each CV, we abstracted the DOM’s current title (permanent, acting, or interim Dean), degrees, medical school, medical specialty, prior leadership positions, and years of appointment into these positions. 10% of CVs were re-coded by a blinded co-author to ensure accuracy in data abstraction. Using publicly available information, the authors assigned demographic characteristics of gender (binary) and race/ethnicity (Black, non-Black Hispanic, Asian, or non-Hispanic White, with each individual assigned to only one group in the order described), as well as each dean’s 2020 US News & World Report Medical School Research ranking (top-25 vs. non-top-25), membership and year of appointment within the National Academy of Medicine (NAM), and Hirsch (H)-index as of October 2019 [14]. Given the sensitive and personally identifiable nature of this information, all data were stored in a password-protected repository within the University of Michigan’s healthcare information technology system and are presented only in the aggregate in this report.

We then evaluated progression through the academic pipeline and common pathways to Deanship using standard descriptive statistics. Notably, we only included those experiences and positions held prior to first DOM appointment in this analysis, and for DOMs without an MD degree, we considered their primary doctorate to be “MD equivalent” for purposes of analysis. Results were then compared using the Fisher’s exact test for categorical data and Wilcoxon rank-sum for continuous data to assess differences by gender and medical school rank. Limited sample size precluded multivariable analyses.

Finally, the authors attest that this research meets all applicable standards for the ethics of experimentation and research integrity.

## Results

### Survey response and demographics

CVs were collected for 95/153 DOM (62%), 5/95 CVs collected were publicly available online. As shown in **Table 1**, this was a representative cohort with the proportion of respondents who were women (15/95, 16%), from top-25 schools (16/95, 17%), and from groups other than non-Hispanic White (15/95, 16%) closely reflecting that of the entire population of invited DOM (16% women, 16% top-25, and 15% from racial/ethnic groups other than non-Hispanic White). For those DOM whose CVs were collected online (n = 5), 20% were female, 20% were from top-25 institutions, and 20% were from groups other than non-Hispanic White, similarly reflecting the overall population. The vast majority of DOMs (94%) in our sample were in permanent DOM positions.

**Table 1:** Characteristics of Medical School Deans.

	All Deans (N = 95)		Male Deans (N = 80)		Female Deans (N = 15)		*P*-value	Top-25 Deans (N = 16)		Non Top-25 Deans (N = 79)		*P-Value*
	N	%	N	%	N	%		N	%	N	%	
**Type of Current Dean Position**												
Permanent	89	94%	75	94%	14	93%	~1	16	100%	73	92%	0.65
Interim	5	5%	4	5%	1	7%		0	0%	5	6%	
Acting	1	1%	1	1%				0	0%	1	1%	
**Race/Ethnicity**												
Non-Hispanic White	80	84%	68	85%	12	80%	0.37	14	88%	66	84%	0.77
Black	6	6%	4	6%	1	7%		1	6%	5	5%	
Non-Black Hispanic	5	5%	3	4%	2	13%		0		5	6%	
Asian	4	4%	4	5%	0			1	6%	3	4%	
**Medical School Rank of Current Institution**^**#**^												
Top-25	16	17%	15	19%	1	7%	0.45	-	-	-	-	
Non Top-25	79	83%	65	81%	14	93%		-	-	-	-	
**Gender**												
Male	-	-	-	-	-	-	**-**	15	94%	65	82%	0.45
Female	-	-	-	-	-	-		1	6%	14	18%	
Year of MD* **Degree**												
< 1970	3	3%	3	4%	0		0.96	1	6%	2	3%	0.59
1970–1979	39	41%	33	41%	6	40%		8	50%	31	39%	
1980–1989	43	45%	36	45%	7	47%		6	38%	37	47%	
> 1990	10	11%	8	10%	2	13%		1	6%	9	11%	
**Year Appointed to First Dean Position**												
1990–2009	24	25%	21	26%	3	20%	0.75	8	50%	16	20%	**0.024**
2010–2019 (within last 10 years)	71	75%	59	74%	12	80%		8	50%	63	80%	
# Years from MD* **to First Dean Position**												
Mean (SD)	31.4	6.2	31.5	6.2	31.1	6.5	0.98	31.1	5.9	31.5	6.3	~1
Median (IQR)	32	27.5–35	32	27.8–35.0	31	26–35		32.5	25.5–35	32	28–35	
**Appointment to First Dean Position**												
Internal	49	52%	40	50%	9	60%	0.58	10	62.5%	39	49%	0.42
External	46	48%	40	50%	6	40%		6	37.5%	40	51%	
**Year Appointed to Current Dean Position**												
1990–2009	17	18%	16	20%	1	7%	0.29	6	38%	11	14%	**0.036**
2010–2019 (within last 10 years)	78	82%	64	80%	14	93%		10	63%	68	86%	
# Years from MD* **Degree to Current Dean Position**												
Mean (SD)	32.3	5.8	32.2	5.8	32.9	5.9	0.59	31.9	5.0	32.4	6.0	0.81
Median (IQR)	33	28.5–35.5	32.5	28.8–35.0	34	29.5–38.0		32.5	29.5–35	33	28.5–36	
**Appointment to Current Dean Position**												
Internal	41	43%	34	43%	7	47%	0.78	7	44%	34	43%	~1
External	54	57%	46	57%	8	53%		9	56%	45	57%	
Additional Degree Beyond MD* **(Master’s or Higher)**												
No	60	63%	50	63%	10	67%	~1	11	69%	49	62%	0.78
Yes	35	37%	30	38%	5	33%		5	31%	30	38%	
PhD or ScD or JD	14	15%	13	16%	1	7%		4	25%	10	13%	
Master’s (e.g. MPH, MBA, MS, etc.)	24	25%	20	25%	4	27%		1	6%%	23	29%	
**H-index**												
Mean (SD)	40.2	28.4	42.3	29.4	28.9	19.2	0.15	73.1	32.3	33.5	22.5	**<0.0001**
Median (IQR)	42	17–50	43.5	18.8–51.0	28	11.5–45.5		68	49.8–89.8	30	15–48	
**Member of National Academy of Medicine Prior to First Dean Position**												
No	73	77%	62	78%	11	73%	0.74	7	44%	66	84%	**0.002**
Yes	22	23%	18	23%	4	27%		9	56%	13	16%	
**Academic Leadership Experience Prior to First Dean Position**												
Permanent Department Chair	69	73%	61	76%	8	53%	0.11	14	88%	55	69%	0.22
Associate Dean	48	51%	38	48%	10	67%	0.26	6	38%	42	53%	0.28
Both Permanent Department Chair and Associate Dean	32	34%	27	38%	5	33%	~1	5	31%	27	34%	~1
Permanent Department Chair without having been Associate Dean	37	39%	34	43%	3	20%	0.15	9	56%	28	35%	0.16
Associate Dean without having been Permanent Department Chair	16	17%	11	14%	5	33%	0.12	1	6%	15	19%	0.29
**Medical Specialty**°												
Internal Med/Family Med/Pediatrics	57	60%	50	63%	7	47%	0.44	12	75%	45	57%	0.40
Surgical^^^	24	25%	19	24%	5	33%		2	13%	22	28%	
Other°	14	15%	11	14%	3	20%		2	13%	12	15%	

Data analyzed by χ^2^ for categorical data (presented as n (%)), and two-tailed t-test for continuous data (presented as mean (SD) or median (IQR)).

^**#**^Top-25 research institution according to 2020 US News & World Report Research Ranking

*MD, DO, or (for those Deans without Medical Degree), PhD

^@^Including vice, associate, executive, or deputy positions

^Including general, thoracic, vascular, neurosurgical, obstetrics & gynecology, ophthalmology, plastics, colorectal, urologic, otolaryngology, and pediatric surgery

°Including psychiatry, emergency medicine, anesthesiology, neurology, pathology, and biochemistry

### Progression through the pipeline

**Fig 1:**
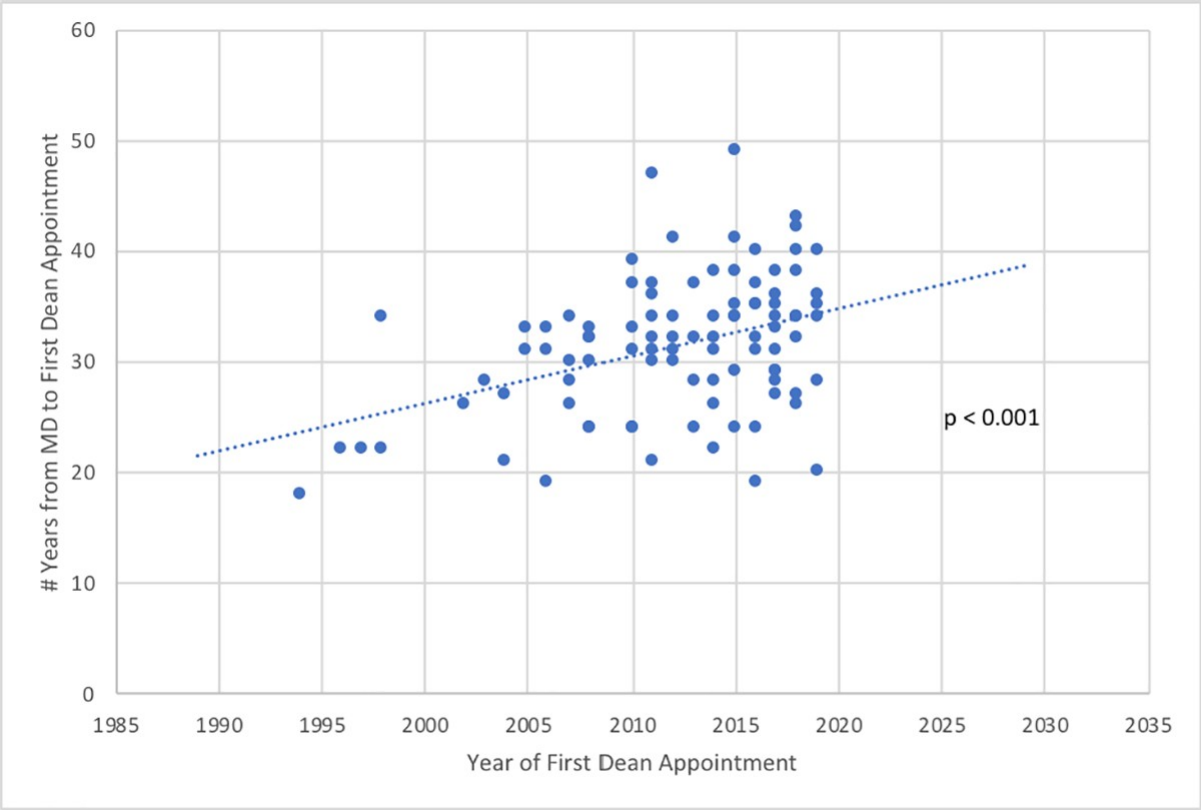
Progression through the pipeline. This figure depicts data from a study of Deans of U.S. Schools of Medicine (95 of 153 total whose CVs were available for review). On average, it takes 31.4 years for an individual to progress from their MD degree to their first Dean of Medicine position. In more recent years, however, it has taken longer to reach Dean of Medicine than it did previously (F-test from general linear model, p < 0.001).

In our sample, 86% of DOMs obtained their MD (or equivalent) degree in the 1970s-1980s. The average (SD) number of years from achieving their MD degree to promotion to their first DOM position was 31.4 (6.2) years. In more recent years, it has taken longer to reach DOM than it did previously (F-test from general linear model, p < 0.001; see **Fig 1**), suggesting either decreased turnover or increased expectations of experience for more recently appointed Deans.

#### A common pathway to Dean of Medicine

As shown in **Table 1**, approximately half of responding Deans were promoted to their first DOM title from internal positions (52%). The most common leadership positions held prior to DOM were Permanent Department Chair (73%), Associate, Vice or Executive Dean (51%), Medical Director (51%), Division Chief (47%), Program Director (38%), and Center Director (35%). Approximately one-third of respondents (34%) had served in both Permanent Department Chair and Associate Dean positions prior to their first appointment as DOM. In addition, 37% of Deans had an additional Master’s level or higher degree at the time of first DOM appointment, and the mean (SD) H-index among the entire cohort was 40.2 (28.4) at the time of survey.

Notably, eight DOMs in our sample had never held Department Chair, Medical Director, or Associate Dean positions prior to their first appointment as DOM. Of these, 7/8 were men and the average number of years from MD degree to appointment was slightly less than the overall cohort (30.3 years). All eight of these individuals had experience as either Division Chiefs or Section Chiefs within their departments, which were largely primary-care (internal medicine, family medicine, or pediatric subspecialty) based.

### The influence of gender on pathways to deanship

As shown in **Table 1**, 15/95 responding DOMs were women (16%). There were no significant differences in the progression of women through the pipeline compared to the overall cohort by era of their MD degree (p = 0.96), year appointed to first or current DOM position (p = 0.75 and 0.29, respectively), or length of time from their MD degree to these positions (p = 0.98 and 0.59, respectively). There might be trends for men to be more likely to serve as permanent Department Chair (76% vs. 53%, p = 0.11) and less likely to serve in Associate Dean (48% vs. 67%, p = 0.26) positions, but these differences were not statistically significant. Within Associate Dean positions 29% of men and 20% of women held Clinical/Business appointments (11/38 vs 2/10, p = 0.71), 45% of men vs 50% of women held Education/Faculty or Student Affairs/Development deanships (17/38 vs 5/10, p~1), and 16% of men and 20% of women held Research positions (6/38 vs 2/10, p = 0.67).

**Fig 2:**
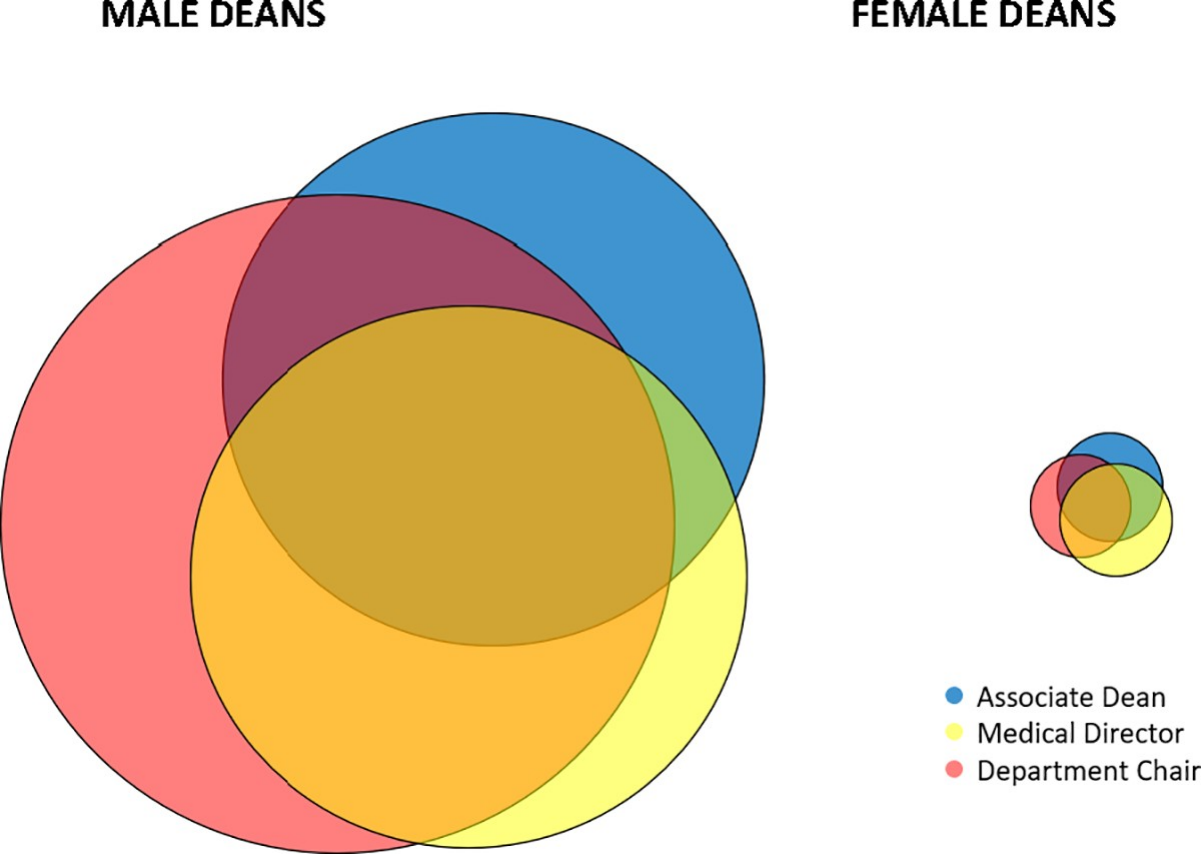
Leadership experience prior to becoming Dean by gender. This figure depicts data from a study of Deans of U.S. Schools of Medicine (95 of 153 total whose CVs were available for review). The three most commonly held leadership positions prior to an individual’s first appointment as Dean of Medicine were Department Chair (73%), Associate Dean (51%), and Medical Director (51%). These area-proportional Venn diagrams, depict prior experience by gender.

**Fig 2** depicts the three most commonly held leadership positions prior to appointment as Dean of Medicine according to gender.

### Experiences and pathways taken by Top-25 Deans

**Fig 3:**
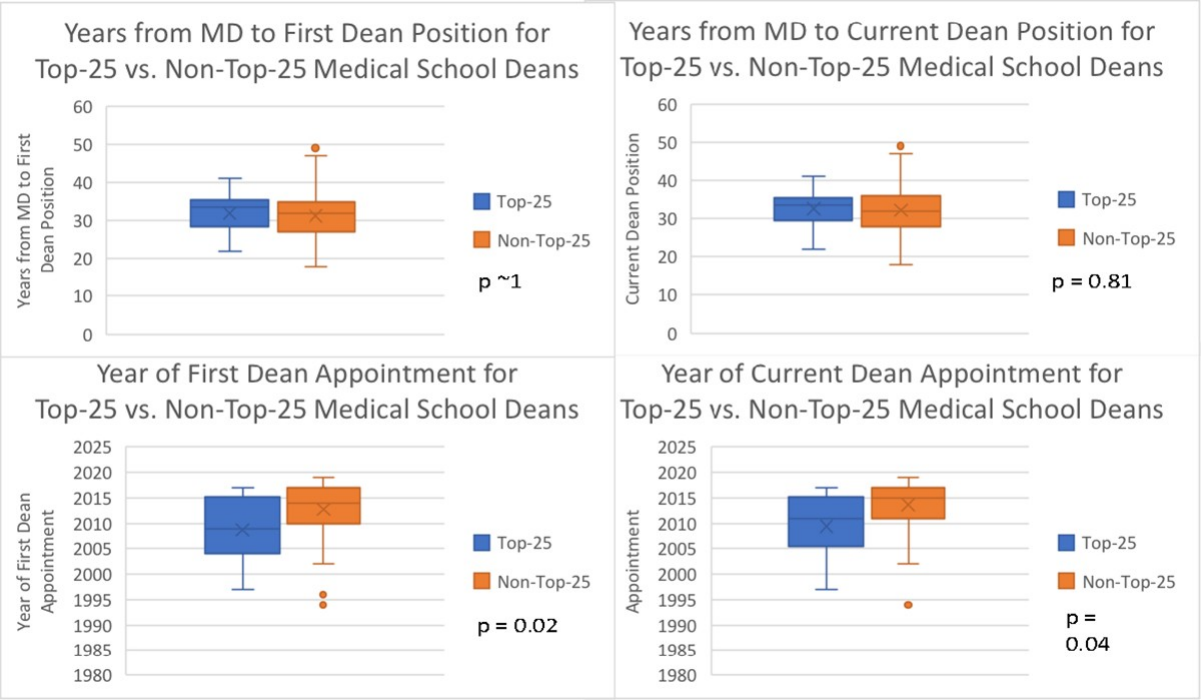
Progression through the pipeline according to medical school rank. This figure depicts data from a study of Deans of U.S. Schools of Medicine (95 of 153 total whose CVs were available for review). When comparing Deans at Top-25 Medical Schools based on U.S. News & World Report’s 2019 research school ranking (top row), there were no significant differences in the number of years between achieving their MD degree to promotion to either the first or current Dean of Medicine position. There were differences in the era of DOM appointments, however, with Top-25 Deans being more likely to have been appointed to both their first and current DOM positions at early timepoints than non-Top-25 Deans (bottom row, p = 0.02 and 0.04, respectively).

**Fig 4:**
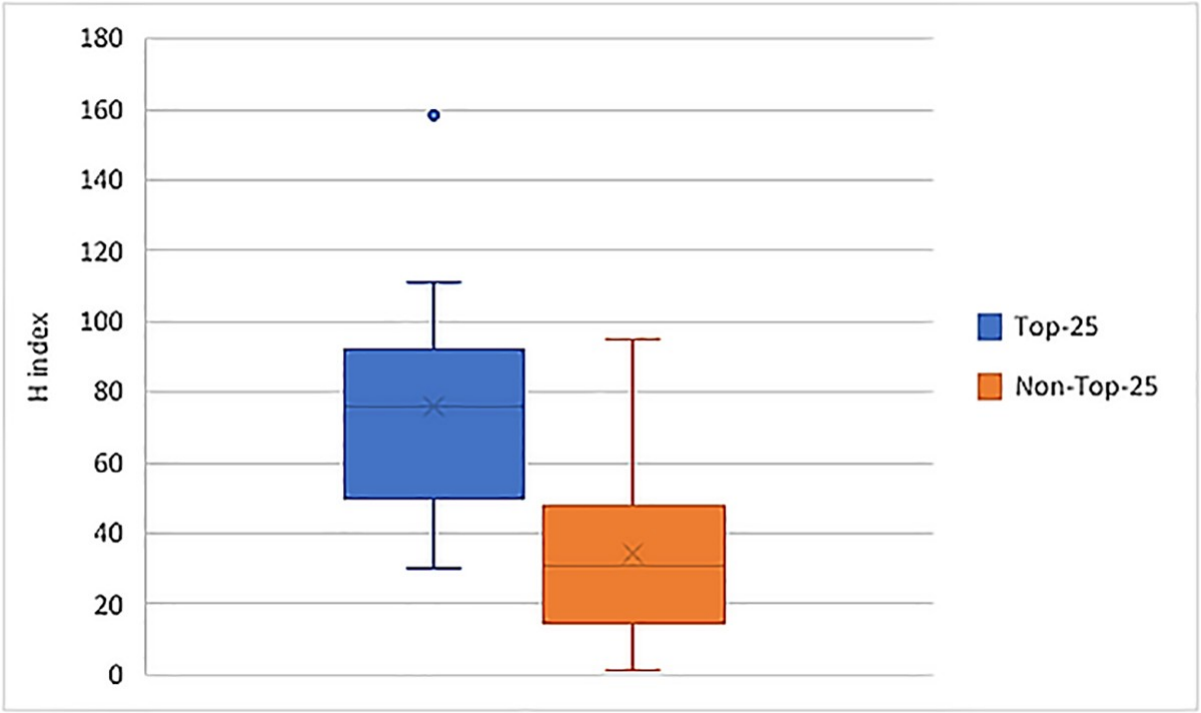
H-Index for Deans of Medicine for top-25 vs. non-top-25 medical schools. This figure depicts data from a study of Deans of U.S. Schools of Medicine (95 of 153 total whose CVs were available for review). When comparing Deans at Top-25 Medical Schools versus Deans of other schools, based on U.S. News & World Report’s 2019 research school ranking, Deans of Top-25 research-ranked medical schools had significantly higher H-indices (mean (SD): 73.1 (32.3) vs. 33.5 (22.5), t-test, p<0.001) than non-Top-25 Deans.

As shown in **Table 1**, 16/95 responding DOM were from Top-25 research ranked Medical Schools (17%). There were no significant differences according to rank in the number of years it took from achieving their MD degree to promotion to either their first (p~1) or current (p = .81) DOM position. There were differences in the era of DOM appointments, however, with Top-25 Deans being more likely to have been appointed to both their first and current DOM positions at early time points than non-Top-25 Deans (**Fig 3**). In fact, more than one-third (37.5%) of Top-25 DOMs were appointed to their current DOM position more than a decade ago, compared to less than one-sixth of non-Top-25 Deans (p = 0.036). Top-25 DOMs were more likely to have NAM membership (56% vs. 17%, p = 0.002) and higher H-indices than other DOMs (**Fig 4,** mean (SD): 73.1 (32.3) vs. 33.5 (22.5), p<0.0001). Of the Top-25 DOMs with NAM memberships, all (7/7) were male.

### Pathways of racial and ethnic minority Deans

An unplanned analysis of the pathways taken by Deans who appeared to be from racial/ethnic groups other than non-Hispanic White (racial/ethnic minority deans) was performed, given their striking underrepresentation both within our responding cohort (15/95, 16%) and among the population of DOM more generally (15%) (**Table 1**). The median H-index of responding racial/ethnic minority Deans was greater, though not significantly different, than that of the non-Hispanic White Deans (47 vs. 40.0, p = 0.42). A similar proportion had an additional degree beyond MD (33.3% vs. 37.5%, p~1) and were appointed into the NAM (26.7% vs. 22.5%, p = 0.74) prior to their first DOM position. It appears that responding racial/ethnic minority DOMs followed similar pathways to Deanship as the entire responding cohort, with 12/15 (80.0%) serving as a Permanent Department Chair, 9/15 as Associate Dean (60.0%), and 7/15 in both positions (46.7%) compared to 71.3%, 48.8%, and 31.3% among non-Hispanic White deans, respectively; all p > 0.56.

## Discussion

This study demonstrates that the types of leadership experiences and achievements shared by individuals prior to their first appointment as DOM reflect the core values and tripartite mission of medicine: excellence in clinical, research, and educational domains. On average, Deans had 30 years of experience before ascending to their position, approximately one-third held additional advanced degrees, and their average H-index was 40, together exemplifying a strong commitment to all aspects of academic medicine as would be expected by its highest leaders. Furthermore, we have elucidated a common pathway to DOM through Department Chair and/or Associate Dean positions, which appropriately reflects the escalating leadership responsibilities and administrative scopes expected of these individuals as they ascend through the clinical, departmental, and ultimately institutional domains of academia.

Our study demonstrated significantly higher H-indices and longer ongoing terms for DOMs at Top-25 research ranked Medical Schools compared to non Top-25 Medical Schools. Unfortunately, despite an excellent response rate, the limited sample size of DOMs overall and included within this study precluded multivariable analyses evaluating the interaction of these variables with one another or with gender. It has already been established that H-index, for instance, is an imperfect measure of scholarly merit that is influenced by publication productivity early in one’s career [15], self-citations [16], co-author circles, and journal prestige, all of which might introduce gender bias based on known differences in early career work-life integration [17, 18], mentorship circles, self-promotional behaviors [19], and gender publication biases [20], particularly within high-impact medical journals [21]. Given the influence that these Top-25 DOMs exert over the mission and funding of medical research within the U.S. as whole, we encourage consideration of how prioritizing certain criteria might contribute to the dearth of women and minority voices, ideas, and heuristics among this cohort and how this might adversely impact the ability of the profession to serve its mission in the long-run [13].

The findings of our study are consistent with prior reports suggesting that both Department Chair and Associate Dean positions are common stepping stones to DOM [1, 2]. There were no differences by gender in the number of years of clinical experience, additional advanced degrees, membership within the NAM (which serves as a surrogate marker of academic excellence and reputation), or in the breadth of leadership positions held prior to their first DOM appointment. This evidence stands against common concerns that women are promoted to leadership roles based not on merit, but rather a desire to achieve superficial gender parity [22].

So, why haven’t more qualified women been promoted to DOM? One hypothesis is that women are diverted into decanal positions that do not directly track towards DOM, such as those focusing on education, mentorship, and institutional public image as opposed to corporate strategy, policy, or finance [2]. The AAMC recently reported that women represent 47% of Associate Deans and 52% of Assistant Deans, yet only 18% of DOM [23]. Additionally women are most represented in offices for diversity, equity and inclusion, faculty affairs/development, and student affairs/admissions than research and clinical leadership positions [23]. This is consistent with our data, which demonstrated a possible trend for more women DOMs to have held Associate Dean positions than men prior to their first DOM appointment, although this finding was not statistically significant.

We also observed a non-significant trend for men to have previously served as permanent Department Chairs prior to their appointment (76% vs. 53%). It is well established that men constitute the vast majority (81%) of all U.S. Medical School Department Chairs, and prior evidence showed that their term lengths are, on average, significantly longer than women Chairs’ as well [4, 10]. Indeed, it has been suggested that without the enactment of term limits (as was recently done by the National Institutes of Health) or without diversification of the search pool beyond that of Department Chair, it could take 50 years to reach gender parity among DOM [4, 24]. Furthermore, as Deans serving at Top-25 institutions had significantly longer tenure, without term limits for DOMs at all levels efforts towards diversification will lag even further behind at the highest tier academic institutions.

As mentioned, there are several limitations of this study inherent to its design. Although the demographics of responding DOMs resembled that of the entire pool, there may have been selection bias in survey response such that those Deans who followed more or less alternative pathways to promotion may have felt more or less motivated to participate in this study than others. The assignment of Deans into a binary gender category and a single racial/ethnic identity assigned by the authors rather than by self-identification are also limitations of this study design; although this approach was deliberately designed to allow for inclusion of as many subjects as possible, given that asking for self-reported demographic information from busy DOMs would likely have led to a more limited sample for analysis, it is important to recognize that these categorizations were limited. Additionally, due to the finite number of DOMs and two-thirds response rate, this study is underpowered to evaluate even primary associations between gender and race, let alone the intersection of gender with race and other important variables that might inform future efforts at diversification.

Although our primary study hypothesis related to gender, given the striking underrepresentation of Deans from minority racial/ethnic groups both within our sample and among the entire population of DOM, we also performed an unplanned analysis of the experiences and pathways taken by responding DOMs who appeared to have minority race or ethnicity, as compared to non-Hispanic Whites. It appears that minority Deans followed similar leadership pathways to Deanship, with a high proportion serving in Permanent Department Chair, Associate Dean, or both positions. Although the unplanned nature of this analysis, small sample size, and need to maintain anonymity of responding DOM within our study limit our ability to explore the interaction of race/ethnicity and pathways to academic leadership, this is an important area for future research.

Finally, this study only captured data from those individuals currently serving as DOMs; we did not evaluate pathways of former DOMs or whether there have been changes in selection criteria over time. We urge caution for those aspiring to become DOM to interpret the “common pathways” elucidated in this study as limited necessarily by the population studied; in addition to all of the foregoing limitations, it is important to recognize that we have only collected the success stories.

Despite these limitations, this study represents the most comprehensive cross-sectional attempt to our knowledge that evaluates pathways to becoming DOM. We propose that future efforts would benefit from further describing the motivations that lead some successful academic physicians to pursue deanship over other leadership roles and should seek to determine whether differences in prior experiences and academic achievements, for example, the use of H-index as an imperfect surrogate of scholarly productivity, actually predict for future success in the role of DOM. This particular metric is of great interest, since the achievements necessary to demonstrate great academic impact (as evidenced by H-index) are quite distinct from the outcomes that are expected from a successful Dean. Certainly, the success of a current deanship is not generally understood to be measured as continuation of that individual’s scholarly productivity during their time in the deanship. However, many would agree that a Dean should embody the attributes that one wishes to see reflected by the faculty. That is, appointment of a Dean with an individual history of being a distinguished and impactful scholar is taken as an indicator that they will attract others of scholarly distinction to the institution. This assumption has never been rigorously tested. Objective studies to identify predictors of success in these positions, if identifiable, could then be utilized to refine the *a priori* selection criteria used by institutional search committees to ensure full access to, and equitable evaluation of, those candidates most qualified to lead. For instance, although Department Chairs are certainly apt at the recruitment and retainment of faculty, financial decision-making, and management of issues surrounding workplace culture, these job characteristics and skills can be found among a myriad of leaders within academic medicine, including among Associate Deans, Medical Directors, or Center/Institute Directors. Such knowledge would also enable those aspiring to DOM to gather the requisite experiences, avoid promotions that do not directly lead to deanship, and seize opportunities with the greatest likelihood to benefit their future careers, institutions, and–most importantly–the patients served by academic medicine.

## Supporting information

S1 File Medical schools whose Deans of Medicine were included in the analytic dataset. (DOCX)
