# Supplementary material for: Common pathways to Dean of Medicine at U.S. medical schools
Source: PLoS One. 2021 Mar 25;16(3):e0249078. doi: 10.1371/journal.pone.0249078 (PMC7993860; doi:10.1371/journal.pone.0249078)
Supplement: S1 File — (DOCX) [file pone.0249078.s001.docx]

**Medical Schools Whose Deans of Medicine were Included in the Analytic Dataset**

Boonshoft School of Medicine Wright State University

Boston University School of Medicine

Carle Illinois College of Medicine

Central Michigan University College of Medicine

Chicago Medical School at Rosalind Franklin University of Medicine and Science

Cooper Medical School of Rowan University

David Geffen School of Medicine at UCLA

Donald and Barbara Zucker School of Medicine at Hofstra/Northwell

East Tennessee State University James H. Quillen College of Medicine

Florida International University Herbert Wertheim College of Medicine

Frank H. Netter MD School of Medicine at Quinnipiac University

Geisel School of Medicine at Dartmouth

Geisinger Commonwealth School of Medicine

Georgetown University School of Medicine

Hackensack-Meridian School of Medicine at Seton Hall University

Indiana University School of Medicine

University of Washington School of Medicine

Yale School of Medicine

Jacobs School of Medicine and Biomedical Sciences at the University at Buffalo

John A. Burns School of Medicine University of Hawaii at Manoa

University of Pittsburgh School of Medicine

Kaiser Permanente School of Medicine

Lewis Katz School of Medicine at Temple University

Johns Hopkins University School of Medicine

Loma Linda University School of Medicine

Stanford University School of Medicine

University of Michigan Medical School

Loyola University Chicago Stritch School of Medicine

Marshall University Joan C. Edwards School of Medicine

Medical College of Wisconsin

Meharry Medical College School of Medicine

Morehouse School of Medicine

New York University Long Island School of Medicine

Mayo Clinic Alix School of Medicine

Paul L. Foster School of Medicine Texas Tech University Health Sciences Center

Penn State College of Medicine

Ponce Health Sciences University School of Medicine

Renaissance School of Medicine at Stony Brook University

Rush Medical College of Rush University Medical Center

Rutgers New Jersey Medical School

Saint Louis University School of Medicine

Southern Illinois University School of Medicine

State University of New York Upstate Medical University College of Medicine

Texas A&M University Health Science Center College of Medicine

Emory University School of Medicine

New York University School of Medicine

The Brody School of Medicine at East Carolina University

The Florida State University College of Medicine

The George Washington University School of Medicine and Health Sciences

The Raymond and Ruth Perelman School of Medicine at the University of Pennsylvania

The University of Arizona College of Medicine – Tucson

The University of Texas at Austin Dell Medical School

The University of Texas Health Science Center at San Antonio Joe R. and Teresa Lozano Long School of Medicine

The University of Texas Medical Branch at Galveston School of Medicine

The University of Texas Rio Grande Valley School of Medicine

The University of Texas Southwestern Medical School

The University of Toledo College of Medicine and Life Sciences

Tufts University School of Medicine

Tulane University School of Medicine

Universidad Central del Caribe School of Medicine

University of Chicago Division of the Biological Sciences, The Pritzker School of Medicine

University of Alabama School of Medicine

University of California, San Francisco School of Medicine

University of Arkansas for Medical Sciences College of Medicine

University of California, Irvine School of Medicine

University of Colorado School of Medicine

University of Illinois College of Medicine

University of North Carolina School of Medicine Chapel Hill

University of Kansas School of Medicine

University of Louisville School of Medicine

Columbia University Vagelos College of Physicians and Surgeons

University of Miami Leonard M. Miller School of Medicine

Icahn School of Medicine at Mount Sinai

University of Minnesota Medical School

University of Mississippi School of Medicine

University of Missouri-Columbia School of Medicine

University of Missouri-Kansas City School of Medicine

University of Nebraska College of Medicine

University of Nevada, Las Vegas School of Medicine

University of Nevada, Reno School of Medicine

University of New Mexico School of Medicine

University of North Dakota School of Medicine and Health Sciences

University of Puerto Rico School of Medicine

University of South Carolina School of Medicine, Columbia

University of South Carolina School of Medicine, Greenville

University of South Dakota Sanford School of Medicine

University of Tennessee Health Science Center College of Medicine

University of Utah School of Medicine

University of Wisconsin School of Medicine and Public Health

USF Health Morsani College of Medicine

Virginia Commonwealth University School of Medicine

Virginia Tech Carilion School of Medicine

Wake Forest School of Medicine of Wake Forest Baptist Medical Center

Wayne State University School of Medicine

Western Michigan University Homer Stryker M.D. School of Medicine
